# Acousto–Optic Modulation and Deflection of Terahertz Radiation

**DOI:** 10.3390/ma15248836

**Published:** 2022-12-10

**Authors:** Pavel Alekseevich Nikitin, Vasily Valerievich Gerasimov, Ildus Shevketovich Khasanov

**Affiliations:** 1 Scientific and Technological Centre of Unique Instrumentation RAS, 117342 Moscow, Russia; 2 Department of Physics, Novosibirsk State University, 630090 Novosibirsk, Russia; 3 Budker Institute of Nuclear Physics SB RAS, 630090 Novosibirsk, Russia

**Keywords:** acousto–optic interaction, terahertz radiation, diffraction, liquefied inert gas

## Abstract

It is known that one of the ways to increase the energy efficiency of acousto–optic devices is to use ultrasound beams with a higher power density. It has been established experimentally that the use of a partially electroded ultrasonic transducer significantlyincreases the energy efficiency of the acousto–optic modulator of terahertz radiation. In addition, the operation of an acousto–optic deflector of terahertz radiation with the use of a sectioned ultrasound transducer was theoretically investigated. It showed that a deflector of this kind enables one to achieve higher angular resolution.

## 1. Introduction

The amount of transmitted information increases exponentially every year. The solution to this problem is the use of data compression algorithms as well as the use of signals with higher frequencies. Therefore, the communication development in the terahertz (THz) range is now relevant [1]. There are a number of sources and detectors of THz radiation [2], and a digital signal processing for high-speed THz communications has also made significant progress [3]. The main problem is the lack of devices for effective real-time control of THz radiation. Due to the specificity of this range, it was possible to create fairly simple metamaterials with a negative refractive index [4] as well as the adaptive metasurfaces for deflecting and modulating THz radiation [5,6]. Their optical properties are highly correlated with conductivity changes which can be caused by impact of the laser radiation or electrical signals. The best results have been achieved by graphene-based devices: the modulation depth is close to 100% and the operation speed is approximately kHz.

Another method for radiation control is based on a well-known acousto–optic (AO) interaction. AO devices are widely used for real-time optical processing of information [7]. This is achieved via control of the parameters of the ultrasound wave, which forms a phase diffraction grating in the medium, resulting in high operation speed of the commercial AO devices in the order of MHz. An important feature is that the intensity of the diffracted radiation resonantly depends on the wavelength of the radiation. Therefore, AO devices can be used for spectrometric measurements. For example, in 2020, an AO spectrometer was developed for the ExoMars space mission, which makes it possible to estimate H_2_O content [8]. AO devices can also be used as a phase modulator in frequency-modulation heterodyne spectroscopy, which allows the analysis of both absorption and dispersion properties of optical resonances [9]. Finally, significant progress has been made in AO-modulated diffuse correlation spectroscopy to monitor blood flow in tissues [10].

To work with visible radiation, ultrasound frequencies of about 100 MHz are used [11]. As the ultrasound transducer is an open resonator, its thickness *h* is inversely proportional to the resonance frequency $F_{\mathrm{res}}$ [12]:

(1) $$F_{\mathrm{res}}=\frac{V_{\mathrm{PZT}}}{2h},$$

where $V_{\mathrm{PZT}}$ is the sound velocity in the transducer material. At $V_{\mathrm{PZT}}\approx 5$ km/s and $F_{\mathrm{res}}=100$ MHz, the thickness *h* of the transducer is only 50 *μ*m. Therefore, a transducer with a width *d* of a few millimeters can be considered as thin. As it is known, thin transducers have oscillations of piston type and generate a homogeneous acoustic field.

Meanwhile, when working with THz radiation, it is necessary to take into account the strong divergence, which is inversely proportional to the wavelength λ. Therefore, wide radiation beams with a diameter of about D=1 cm are used [13]. Obviously, with an ultrasound transducer with a width d=1÷2 mm, most of the radiation will not interact with the ultrasonic beam. On the other hand, it is not reasonable to use an ultrasound transducer with a width much greater than *D*, as it leads to a significant decrease in the acoustic power density. Therefore, the optimal width of the transducer should be comparable the diameter of the radiation beam *D*.

The energy efficiency $\xi_{\mathrm{norm}}=\xi /P_{a}$ of the AO modulator is determined as diffraction efficiency ξ per 1 W of acoustic power $P_{a}$ [14]:

(2) $$\xi_{1D}=\frac{I_{1}}{I_{0}}\approx \frac{\pi^{2}}{2\lambda^{2}}\frac{M_{2}P_{a}}{d}L\exp\left(-\alpha_{s}l\right),$$

where index 1D means that it corresponds to a simple 1D model; $I_{1}$ is intensity of the diffracted radiation, whereas $I_{0}$ corresponds to the transmitted radiation; $M_{2}$ is the coefficient of the AO figure of merit of the interaction medium; $\alpha_{s}$ is the sound power attenuation coefficient; *l* is the distance from the sound transducer to the THz beam; and *L* is the length of the ultrasound transducer.

There are several works related to AO in the THz range [15,16,17]. In these works, AO interaction was investigated in the following media: single crystals of germanium (Ge) and gallium arsenide (GaAs), TPX-plastics, and non-polar liquids (saturated hydrocarbons and their derivatives, in which one or more hydrogen atoms are replaced by other chemical elements). However, the AO diffraction efficiency in these media was only a few hundredth of a percent per 1 W of driving electrical power. It has been found in [18] that the best medium for AO diffraction of THz radiation is liquefied sulfur hexafluoride SF_6_. It is characterized by an AO figure of merit $M_{2}$ two orders of magnitude greater than the abovementioned media. However, this medium is characterized by significant attenuation of ultrasound ($\alpha_{s}\approx 1.6$ dB/cm at F=300 kHz and temperature *t* = 21 °C), which is proportional to the square of the ultrasound frequency *F*. Therefore, the frequency has to be about 300 kHz, which corresponds to a transducer thickness of 6 mm. The width (≈10 mm) of such a transducer is comparable with its thickness (≈6 mm), and it can no longer be considered “thin” plate.

Previously, we have performed a series of experiments on the AO diffraction of THz radiation in the liquefied SF_6_ using ultrasound transducers of various widths *d* (from 6 to 14 mm) operating at a frequency of about F≈300 kHz [14]. As a result, the dependence of the energy efficiency $\xi_{\mathrm{norm}}$ on the width *d* of the transducer was determined. According to the formula (2), with reducing the width *d* of the transducer, the diffraction efficiency ξ should increase by the law ξ∝1/d. It was experimentally established that this law is implemented only for relatively wide ultrasound transducers with a width *d* greater than 12 mm. However, for narrower transducers, an unexpected result was obtained: the energy efficiency sharply decreased with a reduction in the width of the transducer at d≤10 mm (see Table 1).

For wide transducers (which can be approximately considered “thin”), the experimental data are consistent with the model, while for narrow transducers (which are “thick”) the data differ from the model. A literature review showed that complex mechanical deformations occur in “thick” transducers, resulting in a complex structure of the ultrasonic field [19,20]. Therefore, we associate the unusual dependence of $\xi_{\mathrm{norm}}\left(d\right)$ in [14] with the inhomogeneity of the acoustic field caused by complex oscillations of the ultrasound transducer.

In [21], it is shown that in order to achieve greater uniformity of the transducer vibrations, only part of its radiating surfaces should be covered with electrodes. Thus, it is expected that this will lead to a significant improvement in performance of the THz AO modulator. Previously, we used ultrasound transducers that were completely covered with electrodes [14]. Now, we have applied the method of increasing the homogeneity of the acoustic field, proposed in [21]. In this paper, we present a more general model of the AO modulation as well as the results of experiments with transducers whose radiating surfaces were partially covered by electrodes.

**Figure 1:**
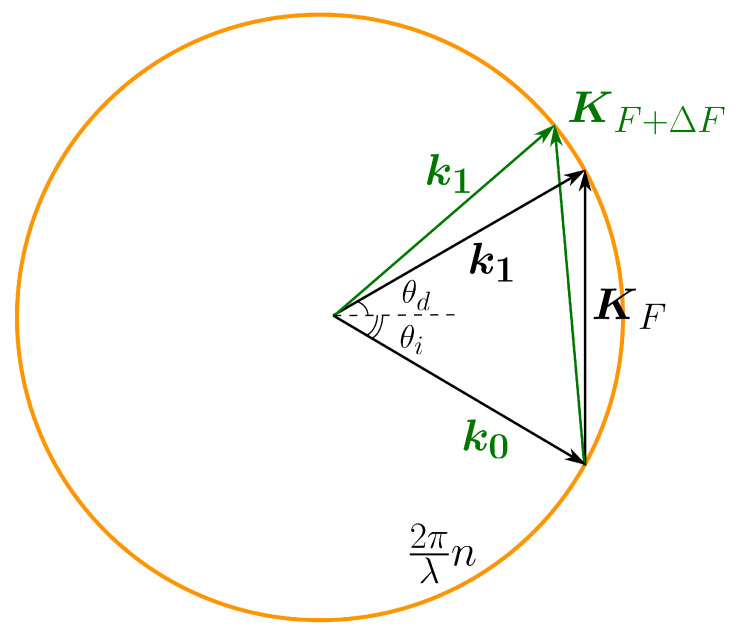
Wave vector diagram of AO diffraction on ultrasound with frequency *F* (black) and F+ΔF (green).

In addition to improving the efficiency of AO modulation of THz radiation, another problem is the implementation of THz AO deflector. For effective AO deflection of THz radiation, the Bragg matching condition (η=0) must be satisfied in a wide range of ultrasound frequency *F* (see Figure 1) [22]:

(3) $$k_{1}=k_{0}+K+\eta ,$$

where η is the mismatch vector, K is the wave vector of the ultrasound, and $k_{0}$ and $k_{1}$ are the wave vectors of the incident and diffracted radiation, respectively.

The mismatch vector η is perpendicular to the boundary of the sound column from the end of vector K to the radiation wave surface [23]. Therefore, as shown in Figure 1, the sound wave vector K must rotate with the frequency *F* so that its end touches the radiation wave surface. In [23,24], it was shown that a sectioned ultrasonic transducer can be used to solve this problem in the visible range. Earlier in [14], an ultrasound transducer completely covered with electrodes was used, which was only suitable for effective modulation, but not for broadband deflection of THz radiation. In this work, we theoretically investigate the operation of the THz AO deflector with a sectioned ultrasonic transducer with the aim of increasing the number of resolvable spots.

## 2. Theoretical Background

### 2.1. THz Acousto–Optic Modulator

**Figure 2:**
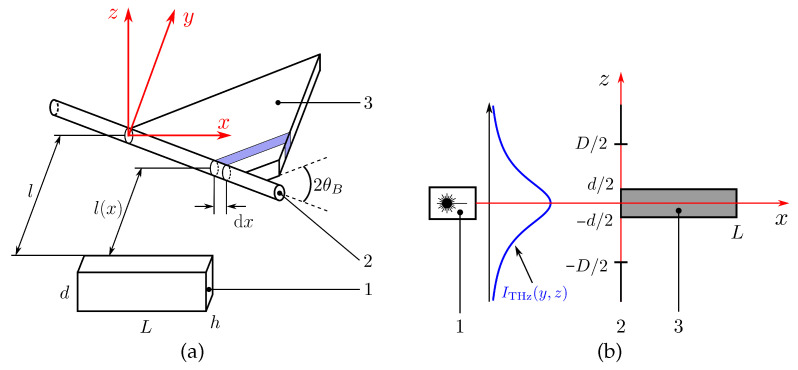
Schematic of AO interaction: (**a**) 1—ultrasound transducer, 2—transmitted radiation beam, 3—diffracted radiation beam; (**b**) 1—radiation source, 2—diaphragm, 3—region of AO interaction.

The AO diffraction efficiency depends on the ratio between the width of the sound column and the diameter of the incident radiation beam (see Figure 2a). Let the width of the sound beam be equal to the width *d* of the region of the sound transducer covered by the electrode and the diameter *D* of the radiation beam be limited by the diaphragm (see Figure 2b).

The intensity distribution over the cross section of the radiation beam for most sources has the form of the Gaussian function:

(4) $$I_{\mathrm{THz}}\left(y,z\right)=I_{s}\frac{1}{\pi W^{2}}\exp\left(-\frac{y^{2}+z^{2}}{W^{2}}\right).$$

The integral intensity $I_{0}$ of the transmitted beam can be calculated by the following relation:

(5) $$I_{0}=\exp\left(-\alpha L\right)\int_{-\infty }^{+\infty }dy\int_{-D/2}^{D/2}I_{\mathrm{THz}}\left(y,z\right)dz=I_{s}\mathrm{erf}\left(\frac{D}{2W}\right)\exp\left(-\alpha L\right).$$

The integral intensity $I_{1}$ of the diffracted beam is limited by the smallest quantity $z_{\max}$ among *d* and *D*:

(6) $$z_{\max}=\left\{\begin{array}{cc} d & \mathrm{for}\;d\leq D;\\ D & \mathrm{for}\;d>D. \end{array}\right.$$

To calculate the intensity of diffracted radiation, we divided the path of the radiation beam into small intervals d*x*. The radiation on each is diffracted in accordance with Formula (2), with d*x* substituted for *L*. The oblique propagation of the radiation leads to a slight change in the length *l* in (2): $l-\tan\theta_{B}x$. Finally, the radiation beam is not infinitely narrow, which must also be taken into account: $l\left(x\right)=l-\tan\theta_{B}x-y$.

Thus, the integral intensity of the radiation diffracted on the ultrasonic field with plane wavefront can be calculated as follows:

(7) $$I_{1}=\frac{\pi^{2}}{2\lambda^{2}}\frac{M_{2}P_{a}}{d}\int_{-z_{\max}/2}^{z_{\max}/2}dz\int_{-\infty }^{+\infty }dy\int_{0}^{L}I_{\mathrm{THz}}\left(y,z\right)\exp\left[-\alpha_{s}\left(l-\tan\theta_{B}x-y\right)\right]dx.$$

The following expression for $I_{1}$ was obtained:

(8) $$I_{1}=I_{s}\frac{\pi^{2}}{2\lambda^{2}}\frac{M_{2}P_{a}}{d}\frac{\mathrm{erf}\left(z_{\max}/2W\right)\exp\left(-\alpha_{s}l+\alpha_{s}^{2}W^{2}/4\right)\left[\exp\left(\alpha_{s}L\tan\theta_{B}\right)-1\right]}{\alpha_{s}\tan\theta_{B}}.$$

Let us estimate the Bragg angle for AO diffraction of THz radiation in liquefied SF_6_. Assume that the frequency of ultrasound is 300 kHz, the speed of sound—V=300 m/s, and the radiation wavelength—λ=130μm. Under these conditions, the Bragg angle is only a few degrees, which corresponds to a quasi-orthogonal geometry of the AO interaction [25]:

(9) $$\theta_{B}\approx \frac{\lambda F}{2V}\approx 4^{{}^{\circ}}.$$

Since the Bragg angle $\theta_{B}$ is much less than unity, Relation (8) can be expanded into the Taylor series:

(10) $$I_{1}=I_{s}\frac{\pi^{2}}{2\lambda^{2}}\frac{M_{2}P_{a}}{d}L\left(1+\frac{\alpha_{s}L\theta_{B}}{2}\right)\;\mathrm{erf}\left(\frac{z_{\max}}{2W}\right)\exp\left(-\alpha_{s}l+\frac{\alpha_{s}^{2}W^{2}}{4}\right).$$

The diffraction efficiency can now be calculated as the ratio of the integral intensity of the diffracted beam to the integral intensity of the transmitted beam:

(11) $$\xi =\frac{I_{1}}{I_{0}}=\left\{\begin{array}{cc} \xi_{1D}\left(1+\frac{\alpha_{s}L\theta_{B}}{2}\right)\exp\left(\frac{\alpha_{s}^{2}W^{2}}{4}\right)\;\mathrm{erf}\left(\frac{d}{2W}\right)/\;\mathrm{erf}\left(\frac{D}{2W}\right)\; & \mathrm{for}\;d\leq D;\\ \xi_{1D}\left(1+\frac{\alpha_{s}L\theta_{B}}{2}\right)\exp\left(\frac{\alpha_{s}^{2}W^{2}}{4}\right)\; & \mathrm{for}\;d>D. \end{array}\right.$$

It follows from (11) that when the electrode width *d* is greater than the diameter *D* of the radiation beam incident on the AO cell, the integral efficiency ξ of AO diffraction decreases in inverse proportion to *d*. This can be explained by the decrease in the acoustic power density, which is inversely proportional to the area of the radiating surface of the ultrasound transducer. At the same time, it should be emphasized that when d<D, the AO diffraction efficiency does not depend on the sound beam width *d*. Note that according to the simple relation (2), the AO diffraction efficiency should be propotional to the acoustic power density. However, as can be seen from (11), this is not the case. This can be explained by the fact that the efficiency of AO diffraction increases, but locally, only in the region of AO interaction, and part of the radiation beam does not interact with the ultrasound. Let us consider an illustrative example. Suppose the initial transducer width *d* is equal to the diameter of the incident radiation beam. If we decrease *d* by a factor of two, the diffraction efficiency in the AO interaction region will increase by the same factor, but only half of the light beam will interact with the sound. Obviously, the influence of these factors is equal but opposite. Together, they balance each other, and the integral efficiency of AO diffraction will not change.

### 2.2. THz Acousto–Optic Deflector

The theory of AO beam steering using a sectioned ultrasonic transducer was developed in [26]. A general expression for the diffraction efficiency ξ was obtained for an even number *m* of the transducer sections. The simplest way to realize AO beam steering is to apply electrical signal to each section with a phase shift π. The operating point $\theta_{i}$ (see Figure 1) of the AO deflector in terms of the angle of incidence was chosen in [26] with the aim of maximizing the number of resolved light spots *N*. However, in that case there was a dip (of 50% of the maximum value) in the center of the frequency response ξ(F). Our task is to choose the operating point $\theta_{i}$ for the diffraction efficiency to be independent on the deflection angle (when the dip in ξ(F) can be neglected).

**Figure 3:**
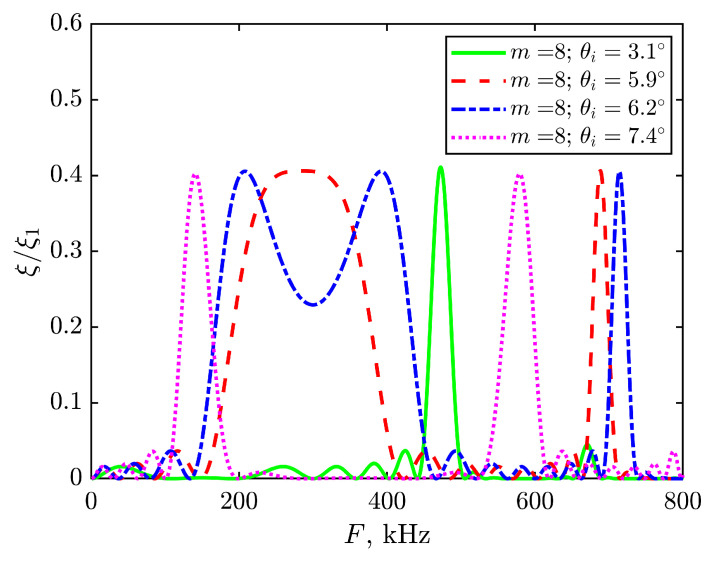
Frequency response of THz AO deflector having an ultrasound transducer with 8 sections for different operating points.

We started with calculating the frequency response for various angles of incidence $\theta_{i}$ in order to reveal the dynamics of the changes in ξ(F). The following values were used: λ=130μm, V=300 m/s, n=1.2, $P_{a}=1$ W, $M_{2}=15,000\times 10^{-15}$ s^3^/kg, L=8 cm, d=1 cm, m=8. The results are shown in Figure 3.

At an arbitrary angle of incidence, the AO diffraction occurs in a narrow frequency bandwidth ΔF (green curve in Figure 3), which limits the number of resolvable spots *N*. However, with a correct choice of operating point $\theta_{i}$, a wide resonance occurs in a low frequency range, which is accompanied by the narrow peak shift to higher frequencies (red curve in Figure 3). A further increase in the angle of incidence leads to a slight shift in the central frequency $F_{d}$ and to the dip presence on $F_{d}$ (blue curve in Figure 3). Finally, the resonance curve splits into two substantially narrower ones (purple curve in Figure 3).

The analysis showed that the AO deflector mode is realized under the following conditions:

(12) $$\begin{array}{cccccc} \theta_{i} & \approx \sqrt{m\frac{\lambda }{nL}};\; & \theta_{d} & =0;\; & F_{d} & \approx V\sqrt{m\frac{n}{\lambda L}}, \end{array}$$

whereas the number of resolved spots *N* depends on the quick action τ=V/D [27] and can be estimated as follows:

(13) $$N=\Delta F\frac{D}{V}=1.9\sqrt{\frac{n}{\lambda L}}D.$$

When using an ultrasonic transducer completely covered with an electrode, the number of resolved spots $N_{1}=1.8nVD/\lambda FL$ is inversely proportional to the AO interaction length *L* [28]. At the same time, according to (13), with a sectioned ultrasonic transducer, $N\propto 1/\sqrt{L}$. Therefore, an AO deflector based on the optically isotropic medium should be equipped with a sectioned ultrasonic transducer. According to our estimates for the parameters given above, for a THz radiation beam with a diameter of 1 cm, the number of resolved spots can be increased up to 3 times: from $N_{1}=2$ to N≈6.5.

## 3. Experimental Results and Discussion

**Figure 4:**
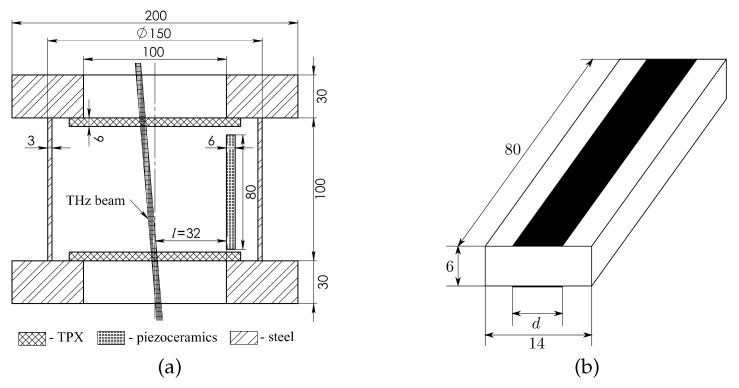
Schematic of THz AO modulator: (**a**) AO cell; (**b**) ultrasound transducer.

For the experimental investigation of the AO modulation of the THz radiation in liquefied SF_6_, a specialized cuvette was used. A set of ultrasound transducers was made of CTS-19 piezoceramics in the form of rectangular parallelepipeds with dimensions of 6 mm × 14 mm × 80 mm (6 mm being the thickness of the transducer, corresponding to a resonant frequency of about 300 kHz). The electrodes were placed on both 14 × 80 mm surfaces of the transducer. The length of the electrodes was L=80 mm, while the width *d* was 6, 8, 10, and 12 mm (see Figure 4).

**Figure 5:**
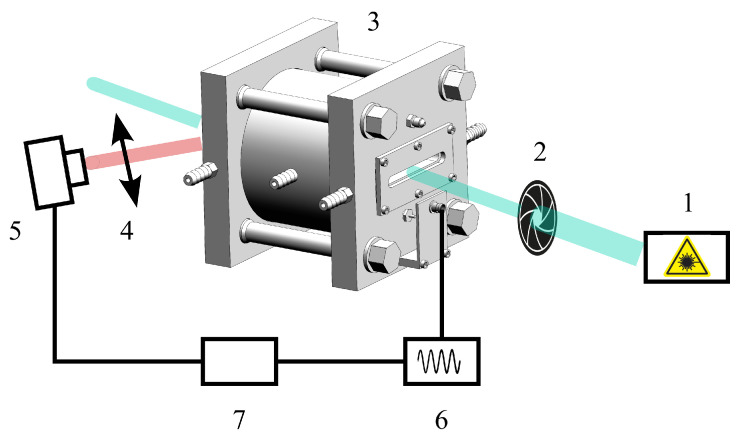
Schematic of experimental setup: 1—radiation source; 2—diaphragm; 3—AO deflector; 4—lens; 5—radiation detector; 6—RF generator; 7—lock-in detector.

**Figure 6:**
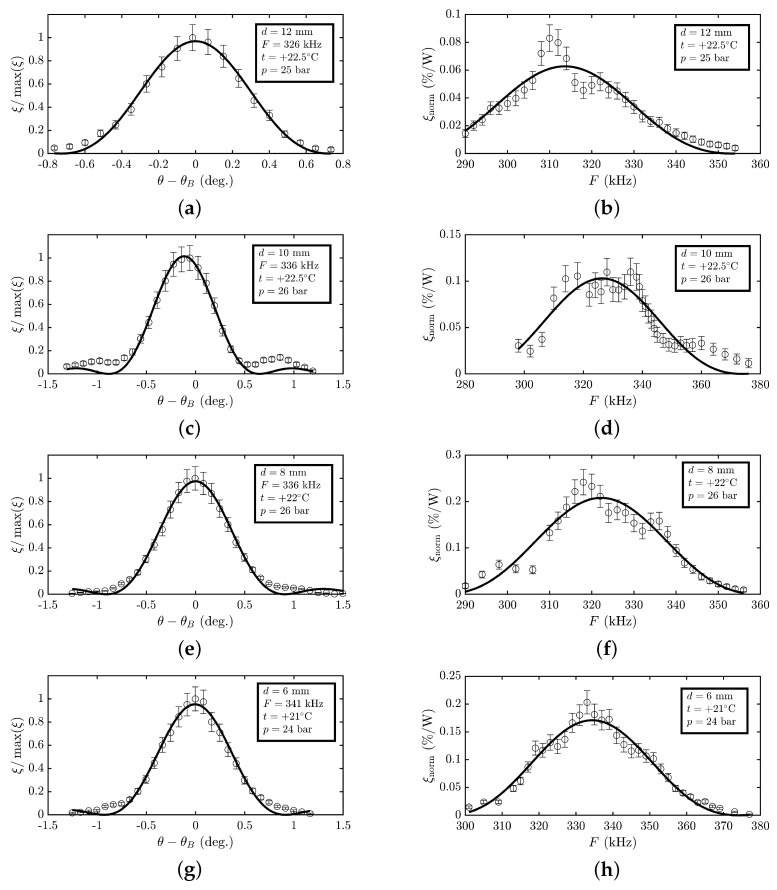
Experimental results: (**a**,**c**,**e**,**g**) AO diffraction efficiency ξ/max(ξ) vs. difference between angle θ of incidence of THz radiation on AO cell and Bragg angle $\theta_{B}$; (**b**,**d**,**f**,**h**) frequency dependence of AO diffraction efficiency per 1 W of applied electric power.

The Novosibirsk free-electron laser (FEL) *1* was used as a source of monochromatic THz radiation with the wavelength λ=130μm (see Figure 5). The THz beam was limited by iris diaphragm *2* with the hole diameter D=7 mm. The radiation deflected by AO cell *3* was focused by lens *4* onto the radiation detector *5* (Golay cell) that has been oriented to achieve the maximum signal. The amplitude of electrical signal with frequency of about F=300 kHz from RF generator *6* was modulated with a frequency 10 Hz. Therefore, the diffracted radiation had the same amplitude modulation increasing the signal-noise ratio, as the Golay cell has the maximum sensitivity at 10–15 Hz. The diffracted radiation intensity $I_{1}$ was proportional to the signal from lock-in detector *7*. Since the diffraction efficiency was about ξ≈0.1%, the mechanical obturator and calibrated optical attenuator (not shown in Figure 5) at the input of the AO cell were employed for determination of the transmitted radiation intensity $I_{0}$. The experimental results are shown in Figure 6.

For each ultrasound transducer, the optimal frequency $F_{\mathrm{res}}$ corresponding to the maximum value $\xi_{\mathrm{norm}}=\xi /P_{a}$ of diffraction efficiency per 1 W of the driving electric power was determined. The data for the angular Δθ and frequency ΔF bandwidth, as well as $F_{\mathrm{res}}$ and $\xi_{\mathrm{norm}}$, are summarized in Table 2. The experimental error in diffraction efficiency $\xi_{\mathrm{norm}}$ was mainly related to the FEL intensity instability and was determined from a 1-minute data sample.

**Figure 7:**
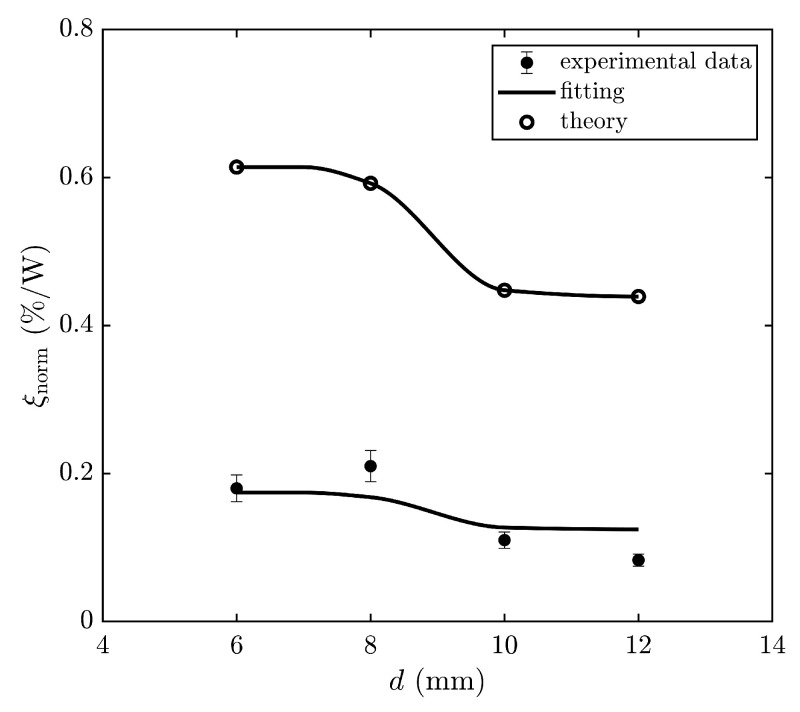
Dependence of energy efficiency of AO modulator on electrode width *d* of ultrasound transducer.

According to (1), the resonance frequency $F_{\mathrm{res}}$ of the sound transducer depends only on its thickness *h*. However, all the transducers had the same thickness h=6 mm and different resonance frequencies $F_{\mathrm{res}}$. This is related to the fact that the transducer width (14 mm) and the electrode width (from 6 to 12 mm) were of the same order as the transducer thickness (6 mm), which resulted in complex deformations. Therefore, a simple model (1) of a piston-type transducer allows only an estimation of the value of the resonance frequency $F_{\mathrm{res}}$. A similar dependence $F_{\mathrm{res}}\left(d\right)$ was revealed in [14] for fully electroded transducers with different widths *d*. The energy efficiency $\xi_{\mathrm{norm}}$ of the AO modulator is proportional to the AO figure of merit $M_{2}$ of liquefied SF_6_, which depends on its pressure *p* and temperature *t*. It should be noted that the experimental conditions were slightly different for each ultrasound transducer. Modelling was performed not for constant *p* and *t*, but for the experimental conditions. Therefore, the calculated dependence $\xi_{\mathrm{norm}}\left(d\right)$ differs from ξ∝1/d (11). The experimental data were fitted by the theoretical dependence reduced by a constant factor (see Figure 7).

The experimentally determined diffraction efficiencies for all the ultrasound transducers were approximately three times lower than that predicted by the theory. So, one can admit the presence of a negative factor (for example, heat losses) in the set of transducers partially covered by electrodes. The physical mechanism can be revealed by modelling complex deformations of the ultrasound transducer. This problem can be solved, for example, in COMSOL multiphysics.

The fitted curve intersects only one of four error bar regions of the experimental data (see Figure 7). Nevertheless, there is a qualitative agreement of the model with all experimental data. At the same time, in [14] the model was valid only for d=14 mm and d=12 mm, whereas for d=10 mm and d=8 mm the difference between the model and experiment was several times, and for d=6 mm this difference was of two orders of magnitude (see Table 1). Therefore, the results of [14] are in fact the first attempt at the experiment, while the current results are in good agreement with the more general theory.

In [14], the diffraction efficiency decreased with a diminishing of the transducer width *d* for d<12 mm. In the current work, we experimentally found that the diffraction efficiency increased with a decrease in the electrode width *d*. For example, for d=8 mm, the diffraction efficiency became two times higher relative to [14], and for d=6 mm, this difference rose to 60 times. The main reason for this difference is the complex structure of the ultrasonic field, as the theory is only valid for an ultrasonic field with plane wavefront. Therefore, we can conclude that ultrasound transducers partially covered by electrodes generate a more homogeneous ultrasonic field and have a great potential in the THz acousto–optics. We also plan to equip the AO cell with the sectioned ultrasound transducer and to experimentally investigate the wide-angle AO deflection. For this purpose, the same electrical signal will be applied to neighboring sections with a phase shift π. This is the simplest design, and according to our estimations, it will enable one to increase the number of resolved spots of the AO deflector by several times.

## 4. Conclusions

The characteristics of the THz AO deflector with sectioned transducer have been estimated for the first time. It was shown that the use of a sectioned ultrasound transducer will increase the number of resolvable spots of the acousto–optic deflector by 3–4 times. It has been demonstrated that the ultrasound transducers partially covered by the electrodes can generate a more homogeneous acoustic field at 300 kHz compared with fully electroded transducers. The AO diffraction in liquefied SF_6_ was investigated employing ultrasound transducers of this type. The diffraction efficiency for an electrode width of 6 mm was 0.18 (%/W), which was two times higher than that for a 12 mm electrode width. Moreover, with an electrode width of 6 mm and transducer width of 14 mm, the diffracted radiation intensity was increased by more than an order of magnitude, compared with fully electroded transducers with the width of 6 mm. This fact enables one to increase the acoustic power density and thus to achieve higher energy efficiency of the THz AO devices.

## Figures and Tables

**Table 1:** Characteristics of the AO modulator of the THz radiation with fully electroded ultrasound transducer obtained in [14].

	d (mm)	14	12	10	8	6
model	$\xi_{\mathrm{norm}}$ (%/W)	0.35	0.40	0.48	0.48	0.48
fitting	$\xi_{\mathrm{norm}}$ (%/W)	0.25	0.28	0.35	0.35	0.35
experiment	$\xi_{\mathrm{norm}}$ (%/W)	0.23	0.31	0.0076	0.11	0.003

**Table 2:** Characteristics of AO modulator of THz radiation revealed experimentally.

*d* (mm)	*t* (°C)	*p* (bar)	$\xi_{\mathrm{norm}}$ (%/W)	Δθ (deg)	ΔF (kHz)	$F_{\mathrm{res}}$ (kHz)
12	+22.5	25	0.08±0.01	0.64±0.03	35±4	313.6±1.4
10	+22.5	26	0.11±0.01	0.68±0.04	42±5	326.3±2.1
8	+22.0	26	0.21±0.02	0.80±0.02	33±3	322.2±1.3
6	+21.0	24	0.18±0.02	0.82±0.03	35±2	334.4±0.8

## Data Availability

The data presented in this study are available on request from the corresponding author. Because of the further research, the data are not publicly available.

## Abbreviations

The following abbreviations are used in this manuscript:

AO	acousto-optic
THz	terahertz
FEL	free-electron laser
